# Model-Based Systems Engineering Applied to Trade-Off Analysis of Wireless Power Transfer Technologies for Implanted Biomedical Microdevices

**DOI:** 10.3390/s21093201

**Published:** 2021-05-05

**Authors:** Juan A. Martínez Rojas, José L. Fernández, Rocío Sánchez Montero, Pablo Luis López Espí, Efren Diez-Jimenez

**Affiliations:** 1 Department of Signal Theory and Communications, Escuela Politécnica Superior, Universidad de Alcalá, Campus Universitario, Ctra. de Madrid a Barcelona km 33.600, 28007 Alcalá de Henares, Spain; rocio.sanchez@uah.es (R.S.M.); pablo.lopez@uah.es (P.L.L.E.); efren.diez@uah.es (E.D.-J.); 2 Independent Consultant, Model-Based Systems Engineering Methodologist, 28807 Madrid, Spain; joseluis.fernandezs@upm.es

**Keywords:** trade-off analysis, medical MEMS, wireless power transfer

## Abstract

Decision-making is an important part of human life and particularly in any engineering process related to a complex product. New sensors and actuators based on MEMS technologies are increasingly complex and quickly evolving into products. New biomedical implanted devices may benefit from system engineering approaches, previously reserved to very large projects, and it is expected that this need will increase in the future. Here, we propose the application of Model Based Systems Engineering (MBSE) to systematize and optimize the trade-off analysis process. The criteria, their utility functions and the weighting factors are applied in a systematic way for the selection of the best alternative. Combining trade-off with MBSE allow us to identify the more suitable technology to be implemented to transfer energy to an implanted biomedical micro device.

## 1. Introduction

New sensors and actuators based on MEMS technologies are increasingly complex and quickly applied to new products. MEMS-based devices demand system engineering approaches, which were previously limited to very large projects, and it is expected that this need will increase in the future, particularly in biomedical applications.

During product research and development, one important step is the powering of the implantable microdevices [1]. For this choice, there are many different technologies that should be analyzed in order to properly select the best option for each product. This trade-off is normally carried out based on previous experience and/or using a classical weighted approach. However, when the trade-off is started from current literature reviews, the amount of data is so large that it becomes necessary to develop more complex and systematic tools for reaching the optimum selection for microdevice applications.

In this work, we propose the application of Model Based Systems Engineering (MBSE) to systematize and optimize the trade-off analysis process. The criteria, their utility functions and the weighting factors are applied in a systematic way for the selection of the best alternative. Combining trade-off studies with MBSE allows us to identify the more suitable technology to be implemented to transfer energy to an implanted biomedical MEMS device. At present, a fully automated algorithm able to perform the complete decision process in the design of a new device is unfeasible. MBSE and trade-off analysis involve a wise combination of science, engineering and art. However, this cognitive endeavor can be made more rigorous by applying certain rules and mathematical techniques. In this work, we show how such techniques are applied to a very difficult challenge, which tries to push the limits of the MEMS engineering to design an implantable medical device with size in the order of 1 mm^3^. Logically, the selected criteria will be severely constrained by this demand.

In Section 2, the systems engineering methodology used in this study is briefly explained. It is a very general, comprehensive and powerful approach, but in this case, we show its application to the trade-off analysis of the best present Wireless Power Transfer (WPT) alternatives for the intended biomedical implantable microdevice.

In Section 3, the state-of-the-art WPT for medical applications is reviewed. In Section 4, we detail the different steps which permit a complete and accurate assessment of different engineering alternatives. This trade-off analysis is described in the context of a larger systems engineering design effort, which is explained in Section 5.

In Section 6, we comment on the results of the trade-off analysis and its implications. The information obtained in this study permits a logical selection of the best WPT system for our application and provides valuable clues to other kind of important studies, such as sensitivity analysis.

## 2. Trade-Off and Heuristics in the MBSE Methodology ISE&PPOOA

At present, there are several MBSE methodologies, more or less complete or general in scope [2]. The ISE&PPOOA methodology is a logically consistent approach to MBSE which combines the best features of the traditional and modern tools for optimal design; for example, N^2^ charts with SysML diagrams. ISE&PPOOA proposes two views of the architecture of the modeled system [3]. These views are the functional architecture and the physical architecture. Below, we briefly describe each of them.

The functional architecture uses diverse SysML diagrams [4] and tables. We understand a function as a transformation to be performed by the system that consumes mass energy or data and generates new ones or transforms them.

Combining diagrams and tables, the functional architecture represents the functional hierarchy using a SysML block definition diagram. This diagram is complemented with activity diagrams for the main system functional flows to represent the system behavior. The N^2^ chart is a table used for an interface description where the main functional interfaces are identified. A textual description of the system functions is provided, as well.

The physical architecture, or architecture of the solution, is developed in two main steps, the results of which are the so-called modular architecture and refined architecture. The modular architecture represents a first version of the solution architecture representing its main logical blocks. These logical block or modules are blocks allocating functions based on the principles of maximum cohesion and minimum coupling between them.

The transition from the modular architecture to the refined architecture is where we apply a combination of trade-off analysis and design heuristics. Although the scope of systems engineering application of trade-off studies is wider, trade-off analysis is prescribed here for choosing and ranking alternative solutions to be applicable to the system component level. Instead of trade-off analysis we recommend the use of design heuristics [3] to implement those non-functional requirements that apply to the architecture design so that they are implemented as design patterns or at the level of system connectors.

Solution architecture is represented by the system decomposition into subsystems and parts using a SysML block definition diagram. This diagram is complemented with SysML internal block diagrams representing the system physical blocks with either logical or physical connectors for each identified subsystem, and activity and state diagrams for behavioral description as needed. A tabular description of the system parts may be provided as well. Functional allocation may be represented either in tabular form or at the system blocks, allocation by definition, or as partitions in the activity diagrams, allocation by usage, represented using SysML notation.

We get benefit of this systematic approach for system engineering to develop an optimal trade-off selection method. In this case, we apply this methodology to WPT technologies for the specific case of an implantable MEMS device. The benefits of integrating the design process for a suitable medical WPT in the range of a 1 mm^3^ into a complete MBSE design framework are manifold, due to its complexity, which demands the exploration of multiple alternatives that have not been already tested for such a small size.

## 3. State-of-the-Art Wireless Power Transfer Technology for Biomedical Applications

Many patients can take advantage of implanted biomedical devices. New microelectromechanical systems (MEMS) technologies allow the development of increasingly complex and miniaturized devices. This reduces many risks associated with the treatment, from surgery to tissue compatibility, due to reduced invasiveness. Another very important factor for implanted devices is energy consumption. Permanent wires inside the body are an unacceptable solution in most cases, while batteries can be dangerous and must be replaced using surgery.

The potential of MEMS for low energy consumption opens the possibility of remote powering without external contacts. The technologies that permit remote powering are collectively known as Wireless Power Transfer (WPT). These technologies are relatively recent and are under development. Although there are many publications exploring detailed aspects of some technologies, there are relatively few reviews comparing different alternative approaches in a systematic and weighted way.

A very recent and complete review was written by Khan et al. in 2020 [5], who compared several WPT technologies using the following parameters: implant type, implant WPT system size, distance from power source to device, type of radiation and frequency, input power, efficiency, test model, SAR (specific absorption rate), safety considerations and technological maturity. The different WPT approaches studied in this article were: NRCC (Non-Radiative Capacitive Coupling), NRIC (Non-Radiative Inductive Coupling), NRMRC (Non-Radiative Magnetic Resonance Coupling), NRRMF (Non-Radiative and Radiative Mid-Field), RFF (Radiative Far-Field), APT (Acoustic Power Transfer) and OPT (Optical Power Transfer). The general conclusion of this review, after a qualitative study of the performance of these technologies, using a Low–Medium–High scale, was that NRIC and NRMRC were better than other WPT techniques due to their moderate size, range and higher PTE performance. Additionally, more complete studies on tissue safety exist for NRIC and NRMRC WPT. APT was comparable to NRIC and NRMRC WPT in terms of performance; the other technologies (NRCC, NRRMF, RFF and OPT) were still too immature from a technological point of view.

Another recent review was provided by Zhou et al. [6], who compared several WPT systems, investigating their key performances such as power transfer capability, power level, efficiency, safety requirements and some others. They organized their review by medical applications, instead of energy types or technologies, but their study was restricted to electromagnetic near-field WPT devices.

Moore et al., in 2019, reviewed the state-of-the-art electromagnetic WPT, especially magnetic resonance, in medicine [7]. They found 17 relevant journal papers and/or conference papers and separated them into defined categories: Implants, Pumps, Ultrasound Imaging and Gastrointestinal (GI) Endoscopy. They found no strong correlation between the system parameters and biomedical applications.

Mahmood et al., in 2019, proposed a new ultrasound sensor-based WPT for low-power medical devices [8]. A 40 kHz ultrasound transducer was used to supply power to a wearable heart rate sensor for medical application. The system consisted of a power unit and a heart rate measurement unit. The power unit included an ultrasonic transmitter and receiver, rectifier, boost converter and super-capacitors. At 4 F, the system achieved 69.4% transfer efficiency and 0.318 mW power at 4 cm. They also compared their work with previous ultrasound WPT systems. They remarked that power and efficiency decreased as the air gap increased to more than 4 cm.

Kakkar in 2018 designed an ultra-low power system architecture for implantable medical devices based on an embedded processor platform chip [9]. This work explored the partitioning of a chip, as well as the trade-offs associated with design choices, especially intelligent power management. He described several design requirements implying ultra-small volume, improved resolution for both sensing and stimulation, integrated electronics design, autonomous operation without batteries and intelligent power management. Concrete values for power operation were provided in figures, being in the order of 250 microwatts.

Shadid and Noghanian, in 2018, wrote a literature survey on WPT for biomedical devices based on inductive coupling, concentrating on the applications using near-field power transfer methods [10]. They compared different systems with the following parameters: frequency, output power, transmitter dimensions, receiver dimensions, gap and efficiency. They plotted efficiency versus delivered power and efficiency versus frequency for the reviewed systems.

Taalla et al., in 2018, presented a comparison of inductive and ultrasonic WPT techniques used to power implantable devices [11]. The inductive and ultrasonic techniques were analyzed studying their sizes, operating distance, power transfer efficiency, output power and overall system efficiency standpoints. They concluded that the inductive coupling approach can deliver more power with higher efficiency compared to the ultrasonic technique, but the ultrasonic technique can transmit power to longer distances.

Agarwal et al., in 2017, presented a comparison of various power transfer methods based on their power budgets and WPT range [12]. Power requirements of specific implants such as cochlear, retinal, cortical and peripheral were also considered. Patient’s safety concerns with respect to electrical, biological, physical, electromagnetic interference and cyber security were also explored. Their conclusion was that EM, NRIC and NCC were better for high-power devices, but for low-power in the order of fewer milliwatts, ultrasonic, mid-field, or far-field technologies are promising.

Dinis et al., in 2017, presented a review of the state-of-the-art implantable electronic devices with wireless power capabilities, ranging from inductive coupling to ultrasounds [13]. They compared the different power transmission mechanisms and showed that the power that current technologies can safely transmit to an implant is reaching its limit. In order to overcome these difficulties, they proposed a new approach, capable of multiplying the available power inside a brain phantom for the same specific absorption rate (SAR) value. They compared previous devices using WPT link distance, antenna/transducer size, received power at the implant, link efficiency and the calculated power density (obtained by dividing the received power by the antenna/transducer size), and ultrasound seemed to be the best WPT solution. Moreover, biological energy harvesters for implantable devices using biologically renewable energy sources, such as muscle movement, vibrations or glucose, were briefly discussed.

The review of Kim et al., published in 2017, focused on Near-Field Wireless Power and Communication for biomedical applications [14]. They proposed that near-field magnetic wireless systems had advantages in water-rich environments, such as biological tissues, due to lower power absorption. However, various issues in near-field magnetic systems remained, such as transmission range, misalignment and limited channel capacity for communications. They suggested that mid-field coupling based wireless powering was convenient for smaller-sized implants using the sub-GHz range. Finally, they indicated that the Q-factor of the coils and their cross coupling are the primary factors that need to be taken into account for system performance optimization.

Lu and Ma (2016) made a review of the best architectures for efficient WPT, allowing further device miniaturization and higher power loss reduction [15]. The main contribution of this article was its best design guidelines for WPT systems based on near-field inductive coupling wireless power transfer. They remarked that operating at a higher WPT frequency can lead to significant size reduction of passive components and better Q values with smaller inductance. However, higher frequencies produced higher tissue absorption inside the human body. They explained that the selection of a correct WPT system architecture for portable or implantable biomedical applications was a trade-off between device volume, efficiency, regulation accuracy, speed and functionality.

Altawy and Youssef, in 2016, studied the trade-off between security, safety and availability in implantable medical devices [16]. They discussed the challenges and constraints associated with securing such systems and focused on the tradeoff between security measures required for blocking unauthorized access to the device and the safety of the patient in emergency situations where such measures must be dropped to allow access. They analyzed the up-to-date proposed solutions and discussed their strengths and limitations.

Safety and thermal aspects of implanted medical devices were described in [17] by Campi et al. in 2016. They studied a WPT system based on magnetic resonant coupling applied to a pacemaker for recharging its battery using a low operational frequency (20 kHz). Other safety considerations for an implantable rectenna for far-field WPT can be found in [18]. Other very informative specific monographs dedicated to WPT for biomedical devices can be found in [19,20,21,22].

All these reviews and monographs articles give exhaustive and detailed information about current trends in WPT. However, even with this information, so many alternatives make it difficult to properly select the most adequate solution for a specific application. Here, we propose a rigorous engineering approach for a trade-off study based on systems engineering and particularly using the models of the system developed by the ISE&PPOOA MBSE methodology. This new comprehensive approach permits conclusion with the most convenient choice and it also allows the ranking of the rest of the solutions for an eventual selection.

## 4. Steps of Trade-Off Studies in the ISE&PPOOA MBSE Context

**Figure 1:**
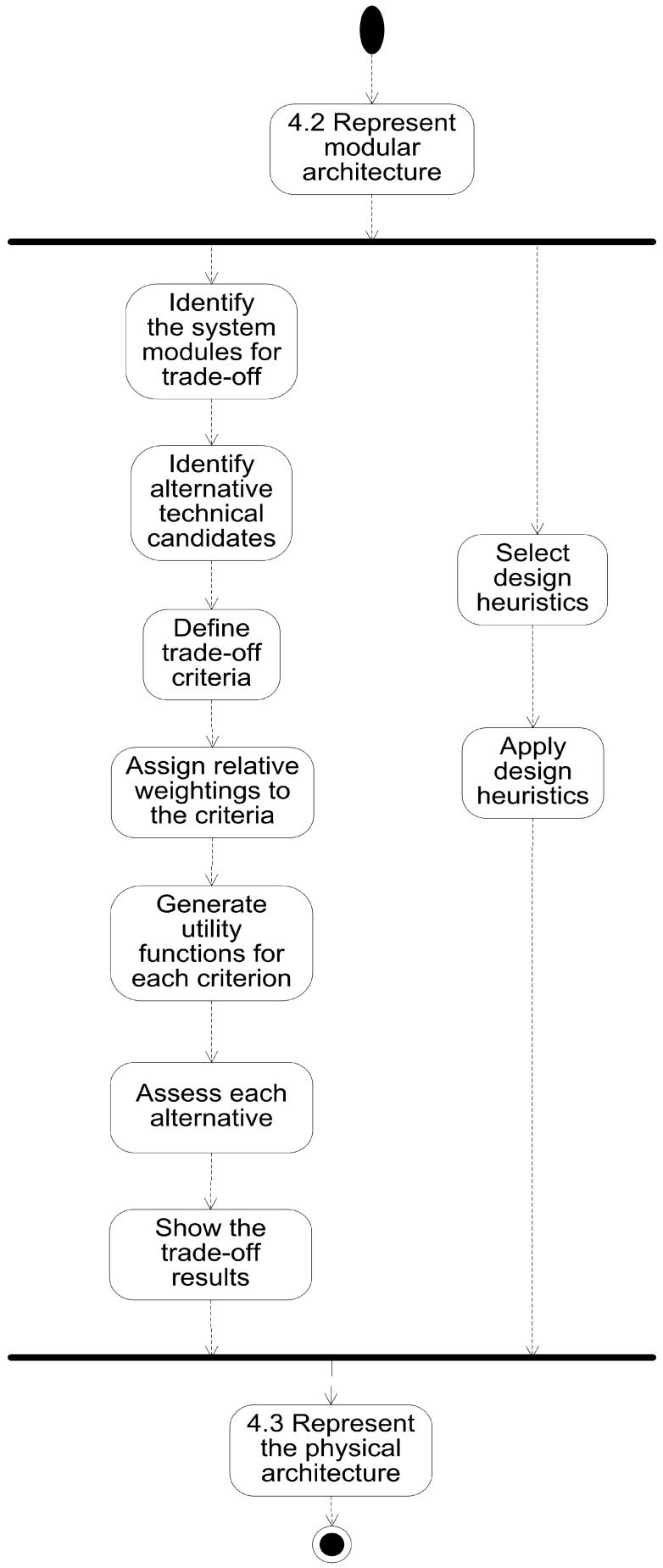
Trade-off and heuristics subprocess for obtaining the refined physical architecture.

Based on diverse processes for trade-off analysis found in the literature, we use here a trade-off analysis process that can be integrated with the ISE&PPOOA architecting design using its outputs and producing inputs to the ISE&PPOOA MBSE process presented in Chapter 4 of the ISE&PPOOA book [3]. Traditional approaches such as those found in the NASA report [23] in 1994 do not use SysML system models. However, recent approaches such as IBM use SysML notation and diagrams [24]. The steps of the proposed trade-off subprocess of ISE&PPOOA are presented in Figure 1.

### 4.1. Identify the System Modules for the Trade-Off Study

From the modular architecture obtained in step 4.2 of the ISE&PPOOA process described elsewhere [3], modules (blocks) are selected, which are the logical building elements clustering cohesive functionality. These may be implemented using alternative technical solutions that are identified in the next step.

### 4.2. Identify Credible Alternative Technical Candidates for Implementing the System Logical Building Blocks or Modules under Consideration

The list of technical alternatives selected during brainstorming sessions may be reduced if the system requirements are considered to have been met [25]. Some alternatives may be discarded based either on cost, technology readiness or other criteria used to eliminate alternatives. The remaining alternatives that need to be assessed should be described in detail.

### 4.3. Define Trade-Off Criteria

Objectives related to stakeholder needs and system requirements are transformed into a set of performance, cost and other criteria to be used for the trade-off study.

### 4.4. Assign Relative Weightings to the Criteria

There are diverse methods for deriving numerical values to the weights to be assigned to the criteria. If a pair-wise comparison is used, we recommend the Analytical Hierarchical Process (AHP) [23] to establish the relative weights to the criteria at the same level, so that all weights sum to 1.0. Another method we recommend and use in the present WPT case is the “swing weight matrix” [26]. Swing weights are assigned to the criteria based not only on importance but on the variation of their scales as well. The reason is that it does not make sense to consider one criterion more important than another without considering the degree of variation among the consequences for the alternatives under trade-off analysis.

The swing matrix is a matrix where the top row defines the value measure importance and the left column represents the range of value measure variation. As recommended by Parnell, weights should descend in magnitude as we move in the diagonal from top left cell to the bottom right cell of the swing weight matrix. Multiple criteria can be placed in the same cell [27].

### 4.5. Generate the Utility Function for Each Criterion

For the trade study, it is necessary to represent, as part of the system model, the selected assessment criterion and the utility or value functions associated to each criterion. Utility functions can be discrete or continuous. Utility functions follow three basic shapes: linear, curve and S shape curve.

For example, when an increasing utility function is created for a particular criterion, the systems engineer ascertains whether the project stakeholders consider it as the minimum value of the measure to be accepted, mapping it to the 0 value on the score scale (*y*-axis). The measure beyond which an alternative provides no additional value is mapped to the highest score scale (*y*-axis). It is important to pick the appropriate inflection points for drawing the curve, which may be either convex or concave.

### 4.6. Assess Each Alternative

Every alternative should be estimated for a given criterion in terms of its score, based on the applied utility function. Then, the relative weights assigned to each criterion are used to compute the objective function that combines the weights and scores. The sum combining function is frequently used. The assessment is based on the created utility functions, using criteria values from each alternative, obtained from test data, vendor provided data, simulations, prototypes, engineering practice or literature.

### 4.7. Show the Trade-Off Results

Generally, a summary table of criteria versus alternatives is presented to summarize the results from the preceding steps. Based on these results, a decision is made. A sensitivity analysis is recommended to determine the robustness of the alternatives selected based on their highest rank in the trade-off analysis [27].

Concurrently, design heuristics are selected and used to refine the architecture. The use of heuristics is recommended to implement non-functional requirements that cannot be allocated to some building blocks but are to be implemented as design patterns or layouts of connectors between the building elements.

## 5. Application of Trade-Off Analysis within the ISE&PPOOA MBSE Approach to Select the Best WPT Alternative for an Implanted Biomedical Device

In this work, we are interested in the design of a challenging WPT system able to power a newly created micromotor for medical intravascular surgery. This demands the previous study of the best technological alternatives for its implementation. Many severe constraints are associated with this problem, including lack of data in the intended size range of 1 mm^3^. Due to this, a comprehensive design framework such as the ISE&PPOOA methodology is needed. We shall study the most promising WPT alternatives to power a 1 mm^3^ MEMS device inside a human artery, using the information reviewed in the Section 3.

Below, we describe the main outcomes produced by performing the trade-off steps described in the previous section.

### 5.1. Identify the System Modules for the Trade-Off Study

**Figure 2:**
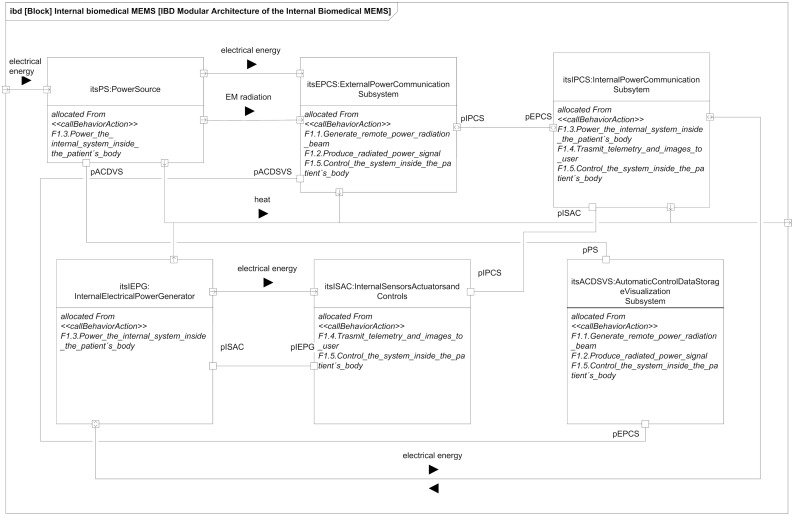
Logical blocks of a biomedical implanted system with WPT.

The modular architecture previously obtained using ISE&PPOOA can be seen as an internal block diagram represented using SysML notation in Figure 2. This architecture was designed for a complex autonomous implanted system with sensors, actuators and communication capabilities. In order to simplify the trade-off study, we limit ourselves to the system parts related to WPT, functionality that can be reduced to the blocks “Power Source” and “Internal Electrical Power Generator”.

### 5.2. Identify Credible Alternative Technical Candidates for Implementing the System Logical Building Blocks or Modules under Consideration

After a comprehensive literature review of the state-of-the-art WPT, summarized in the Introduction, possible candidates to power our MEMS-based device in the one cubic millimeter range are:

- Non-Radiative Inductive Coupling;
- Non-Radiative Magnetic Resonance Coupling;
- Non-Radiative Mid-Field;
- Radiative Far-Field;
- Acoustic Power Transfer.

### 5.3. Define Trade-Off Criteria

Based on the system requirements [25] related to energy transfer and energy harvesting by the device inside the patient body, we select the following trade-off criteria:

- Input power (W), the initial delivered power to the implanted medical device outside the body;
- Power transfer effectiveness (%), the ratio of power produced by the implanted device and the input power;
- Implant WPTRx size (mm), the largest dimension of the implanted medical device;
- Effective operation distance (mm), the maximum distance between the external power source and the implanted device in air (or water for ultrasound) for successful performance;
- Specific absorption rate, SAR (W/kg), the power absorbed per mass of tissue;
- Mechanical complexity (low–medium–high), which depends on the number, size, shape and materials of the system parts. It is also directly related to manufacturing costs. The higher the number of parts, the smaller their size, the more complex their shapes and the costlier their materials, the higher will be the mechanical complexity;
- Technical maturity (low–medium–high), an abstract measure of the degree of consolidation and performance of a technological solution.

Other safety criteria besides SAR and resilience impact the architecture at the connectors level, as well, so we recommend the use of heuristics instead of trade-off studies to implement these safety and resilience requirements.

**Figure 3:**
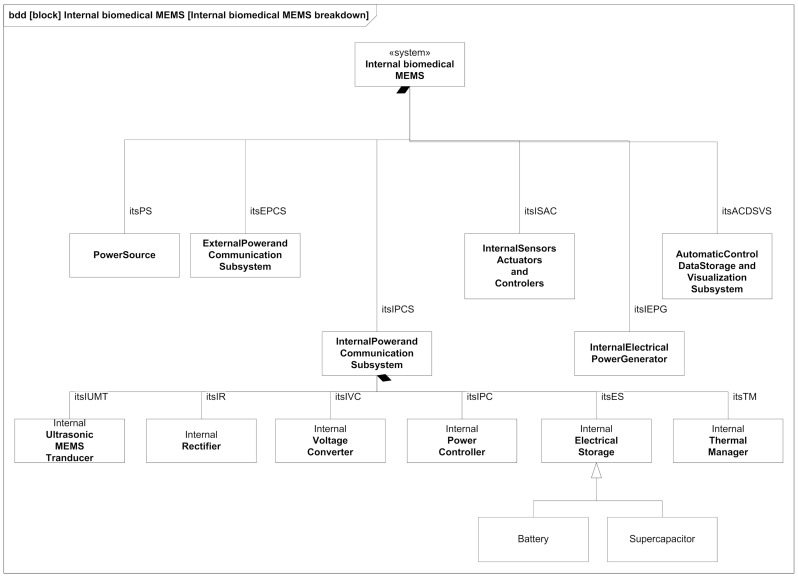
Refined physical architecture of the best WPT alternative (APT) after the trade-off study.

Both the criteria selection process and the application of heuristics cannot be fully automated without human intervention, so a certain degree of subjectiveness, but also creativity, is unavoidable. Additionally, many unknown variables are present in the microdevice design, due to the exploratory and frontier research nature of this project. Thus, the trade-off criteria have been selected trying to constrain the space of alternatives (trade-space) as much as possible in order to produce a reasonable solution for a physical architecture, avoiding the introduction of spurious information that cannot be confirmed at present in the intended range of sizes. Another important consideration in the selection process was that the criteria values obtained from the literature revision could be reasonably extrapolated to our design objectives to build the utility curves. The final result of this process is the refined physical architecture of the best WPT alternative (APT) after the trade-off study, which can be seen in Figure 3.

**Figure 4:**
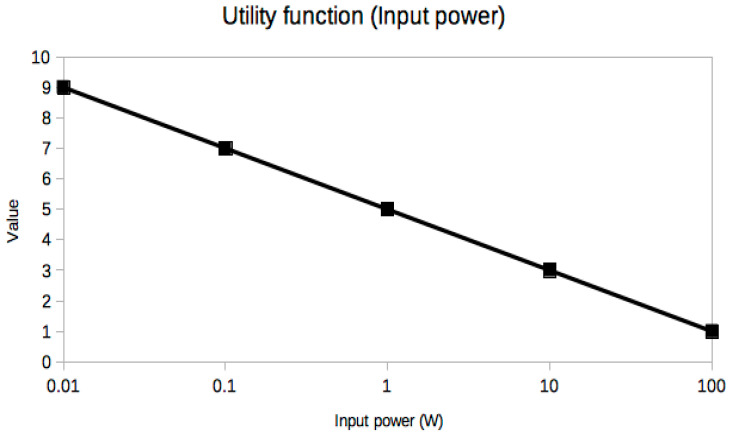
Utility function for Input Power.

**Figure 5:**
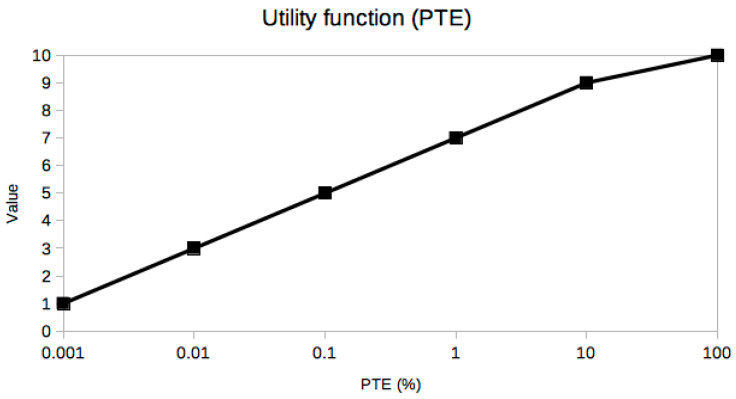
Utility function for Power Transmission Effectiveness.

**Figure 6:**
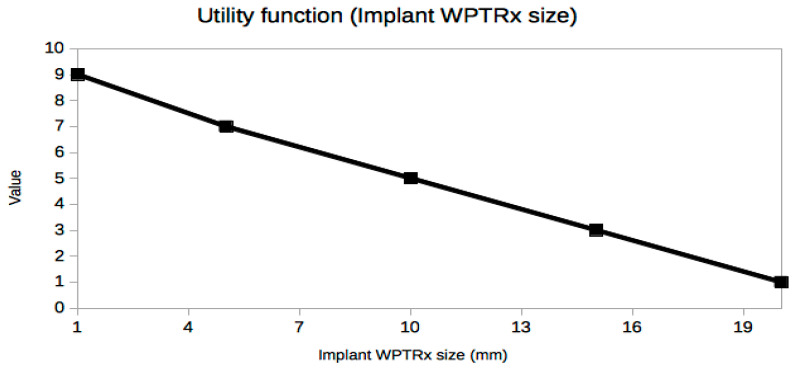
Utility function for Implant WPTRx Size.

**Figure 7:**
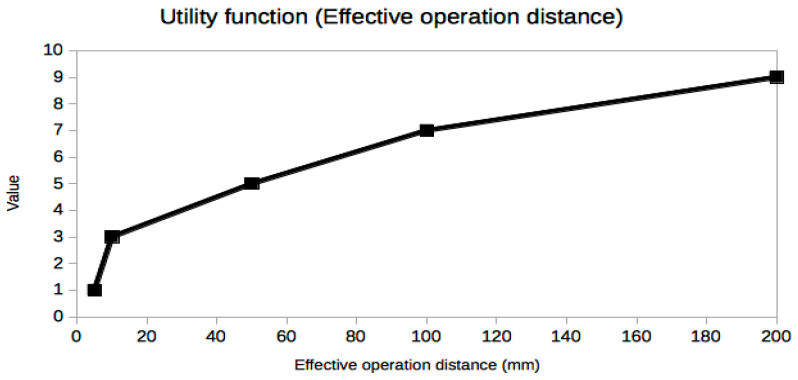
Utility function for Effective Operation Distance.

**Figure 8:**
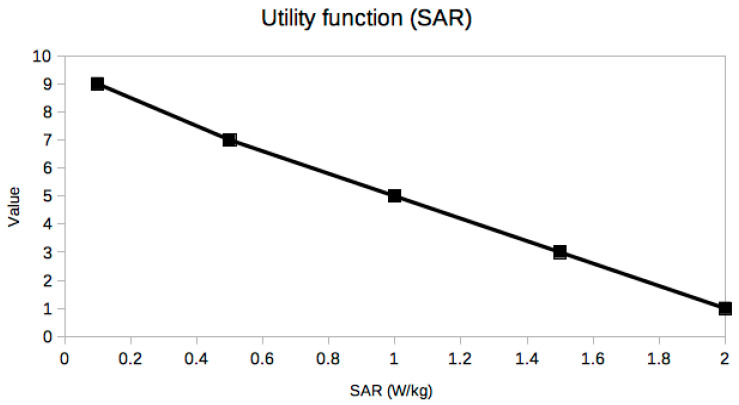
Utility function for Specific Absorption Rate.

**Figure 9:**
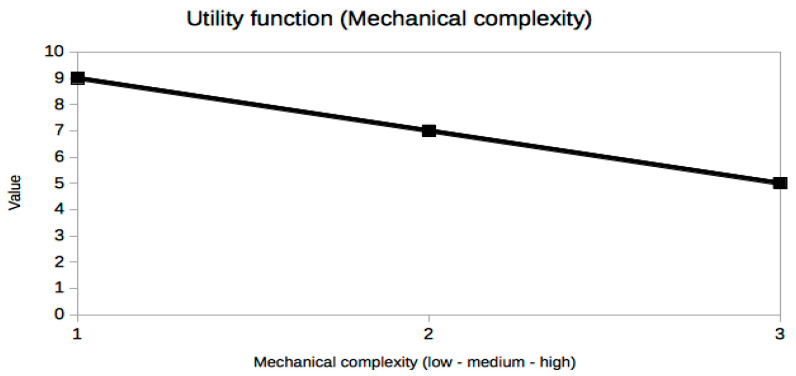
Utility function for Mechanical complexity.

**Figure 10:**
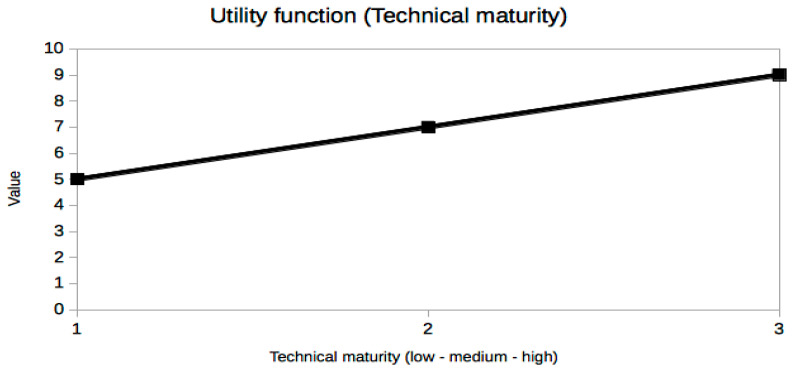
Utility function for Technical maturity.

It is not easy or even possible to know a priori which set of trade-off variables will be optimal for every problem, because it is the result of many constraints, especially the stakeholders’ needs [25], which are usually presented in an ambiguous or less than desirable rigorous way. A very useful piece of advice to confirm that the criteria selection process is correct is based on the exploration of the resulting utility curves. This is a powerful approach to refine the criteria selection process itself in an iterative way, which is inherent to the MBSE design approach. From a mathematical point of view, the curves should be smooth (continuous and differentiable) and restricted to a few reasonable types, such as polynomial, logarithmic or exponential functions. The extremal and inflection points should indicate critical values of the corresponding trade-off criterion. Any lack of regularity, bijectivity (with suitable domain restrictions if necessary, like in exponential or even degree polynomials, for example, in order to be invertible) or oscillations indicate some serious problem with the utility curve and the associated trade-off variable, which must be corrected either by changing the criterion completely or revising the prescribed values. In our case, it can be observed that all utility curves in Figure 4, Figure 5, Figure 6, Figure 7, Figure 8, Figure 9 and Figure 10 fulfill these mathematical conditions. These mathematical-based “metacriteria” for trade-off analysis verification are not explicitly described in the systems engineering literature, as far as we know, and can be considered a valuable contribution.

### 5.4. Assign Relative Weightings to the Criteria

We apply the swing weight matrix for our trade-study because it considers variation in the measured range as well as importance. Thus, the swing weight matrix is more complete than other approaches that only consider importance. The swing matrix obtained for the trade-off criteria selected in the previous step is shown in Table 1. Weights are assigned based on the technical literature review, previous experience with MEMS projects and stakeholders’ needs. One common criticism of trade-off analysis based on weighting criteria outside the systems engineering community is that it involves some degree of subjectiveness. However, this human assessment is unavoidable, because there are many competing stakeholder interests, generally formulated in an incomplete and ambiguous manner, in addition to very complex physical and technical constraints. At present, no algorithm or artificial intelligence approach is able to complete this step automatically without human assistance. Similar observations can be applied to other widely used decision-making approaches such as Analytical Hierarchy Process.

### 5.5. Generate the Utility Function for Each Criterion

Utility functions for the selected criteria can be seen in Figure 4, Figure 5, Figure 6, Figure 7, Figure 8, Figure 9 and Figure 10. These utility functions are built and represented using the recommendations described in ISE&PPOOA book [3] admitting the mathematical conditions described in more detail in Section 5.3.

### 5.6. Assess Each Alternative and Show the Trade-Off Results

The results of this trade-off analysis can be seen in Table 2. Weights have been normalized with respect to the total weight sum of the swing matrix values (weight sum = 4.65), converting the swing weights into measure weights (or global weights), so that their sum is now 1. The final weighted sum for a given technological alternative is calculated as the sum of the products of the measure weights of each criterion by the values obtained from the utility curves for the same criterion. In other words, the values of the weights column are multiplied by the corresponding utility function values of a given alternative column and then summed to produce the final score. Comparing the obtained scores, we can select the preferred alternative. In this case, the Acoustic Power Transfer solution is the best, followed by the Radiating Far Field technology.

## 6. Results and Discussion

The results indicate that the most appropriate technology is APT (Acoustic Power Transfer) due to its transfer effectiveness, size, effective operation distance and SAR. After we identified the technology, we were able to decompose in more detail the system blocks that have to implement it, and we used a BDD diagram made with standard SysML notation (Figure 3).

From the detailed decomposition of these blocks, it is possible to build, by using design heuristics and design patterns, the IBD diagrams that would define the refined architecture of the solution which, for brevity reasons, are not shown here.

Utility curves were obtained compiling relevant data from the revised scientific publications, as shown in Section 3, and verifying its mathematical correctness following the conditions explained in Section 5.3.

Specifically, data for the utility curves were collected from:

Table 2 (page 46) of reference [5];

Table 1 (page 33–34) of reference [7];

Table 1 (page 4) of reference [8];

Table 2 (page 4–5), Table 3 (page 6–7) and Table 4 (page 8) of reference [10];

Table 1 (page 2101) and Table 2 (page 2102) of reference [11];

Table 1 (page 9) of reference [13].

The values correspond to the best results of the state-of-the-art technologies combined. We used a quantitative safety criterion (SAR), but safety heuristics could be applied to refine the architecture as well.

A sensitivity analysis is recommended to determine the robustness of the alternatives. A complete sensitivity analysis is not performed here, but a very useful qualitative analysis can be performed from the information derived from the utility curves, which are far richer in content than their mere use as trade-off tools could suggest.

Input power (Figure 4) is a decreasing linear function in a semilogarithmic scale, which implies that its dynamic range is large. Thus, a small variation in other variables can produce a large variation in the required input power, making this variable very sensitive to small changes in the design for all the studied alternatives. This variable is also one of the most important, so that it can be deduced that the overall performance of the WPT system will be very sensitive. Thus, the input power values can vary for the same alternative with different values of the other variables. Moreover, input power is very relevant from a safety point of view, because it is limited to levels which cannot produce any damage, pain or discomfort on the skin or inside human tissues. Other safety considerations related to input power would be possible electromagnetic interferences and damage to other devices or to human operators.

Power Transmission Effectiveness (PTE) (Figure 5) is a key variable with the largest range of variation of all studied criteria, even larger than the input power; it is the most sensitive value and the latest technological advances are critical to fix it. Its mathematical behavior is an increasing linear function in a semilog scale. For example, ultrasound-based energy harvesters only a few years ago could have a PTE as low as 0.001%, while the most recent ones can achieve 40%. Obviously, such a large increment has changed this alternative from a feasible WPT technology for medical implants to the most promising one in our trade-off analysis for MEMS. Thus, the two most sensitive variables, input power and PTE, have opposite contributions to the performance of the WPT system, because we want the largest PTE with the lowest input power.

Implant size (Figure 6) is one of the most important constraints in our example due to an extreme constraint: it must be fit in a volume of the order of 1 mm^3^. However, PTE decreases exponentially and power input increases exponentially when the device size is decreased, even by small amounts, due to the small range of variation of this variable with a decreasing linear utility function. A sensitivity analysis would reveal that even small building tolerances in the manufacturing process with current technologies could radically change the performance of the final implant, which is unacceptable for a medical device.

The effective operation distance (Figure 7) has a nonlinear response curve. This variable determines the depth of the implant. It can be observed that present technologies do not allow very deep implants in practice if millimeter size devices are desired. The range of variation is small, so that small variations in depth or distance from the external power source or both can produce very large changes in input power and PTE. However, in this case, the sensitivity is even worse than in the case of the device size, because the utility function increase is not linear and the steepest variation can be seen in the smallest distance values, in the range of a few mm. This implies that in order to have a stable power supply, very strict positioning mechanisms should be used, which could be impractical or even unfeasible if the implant can or must be moved.

SAR (Figure 8) is one of the most important safety related metrics, although the input power has to be limited as well. We can see that its range is severely constrained and most of the state-of-the-art devices are dangerously near the limit for the best values of the other variables. Thus, a sensitivity analysis would indicate that even a modest decrease in the SAR values in order to comply with legal restrictions would enormously impact the values of input power and PTE, surely forcing the implant size to be considerably larger, frustrating the goal of 1 mm^3^ total volume if radically new design ideas are not found.

The last two variables, mechanical complexity (Figure 9) and technical maturity (Figure 10), are very different from the previous ones. They are abstract metrics that try to describe the effort and cost of building the WPT system without using economic or monetary values, presently unknown, because we are dealing with a present research project pushing the limits of near-future MEMS technology. In fact, their values are more categorical than numerical, although an easy conversion can be done in order to draw the utility curves, which are simply linear in these cases. In this study, these two variables are not as critical, but within a constrained project budget, much more detailed utility curves should be obtained in this regard. Surely, with accurate costs, their influence would severely constrain the feasible technological options and the conclusions of the trade-off analysis could be very different.

## 7. Conclusions

The combination of MBSE and trade-off analysis is a very useful engineering best practice for the selection of the most suitable technology to be applied to solve a particular problem. Here, MBSE allows us to identify the system level where the application of trade-off should be accomplished. In this way, we identified the system building blocks and which functions should be implemented to solve the problem of a wireless energy transfer and energy harvesting system for a new type of micromotor inside the patient’s body.

The results obtained by this trade-off study are consistent with the results obtained by other researchers and published in the literature, but expand and detail them within the context of a real research systems engineering effort with many unknowns because it pushes the limits of present MEMS technologies to sizes of the order of 1 mm^3^. The methodological approach we propose here helps to inform better design decisions in the early phases of the project, saving costly redesign efforts in later phases. Moreover, we have detailed the mathematical conditions that the utility functions must have in order to be useful for trade-off studies. Finally, we have shown how the information from the trade-off analysis can be used to make a valuable qualitative sensitivity study of the system.

## Figures and Tables

**Table 1:** Swing matrix of the studied criteria. Weights are assigned based on technical literature values and stakeholders needs.

		Level of Importance of the Value Measure		
		Very Important	Important	Less Important
Variation in Measure Range	*High*	Input power: 100 Power transfer effectiveness: 100		
	*Medium*		SAR: 75	
	*Low*	Implant WPTRx size: 90	Mechanical complexity: 50	Effective operation distance: 25 Technical maturity: 25

**Table 2:** Summary of results of the trade-off analysis. Weights are normalized with respect to the total weight sum of the swing matrix values (weight sum = 465).

Swing Matrix Values		Utility Curve Values (for the Best Case)				
Criteria		NRIC	NRMRC	NRMF	RFF	APT
Input power	0.215	9	5	6	9	6
Power transfer effectiveness	0.215	3	7	4	9	9
Implant WPTRx size	0.193	9	9	8	5	9
Effective operation distance	0.054	1	3	5	7	7
SAR	0.161	1	3	5	3	7
Mechanical complexity	0.107	9	7	7	5	5
Technical maturity	0.054	9	9	7	7	5
	Weighted sum	5.981	6.197	5.896	6.609	7.272

## Data Availability

Not applicable.

## References

[1] Kim W.S., Jeong M., Hong S., Lim B., Park S. (2020). Il Fully implantable low-power high frequency range optoelectronic devices for dual-channel modulation in the brain. Sensors.

[2] Estefan J. (2008). Survey of Model-Based Systems Engineering (MBSE) Methodologies.

[3] Fernandez J.L., Hernandez C. (2019). Practical Model-Based Systems Engineering.

[4] OMG Systems Modeling Language. http://www.omgsysml.org/.

[5] Khan S.R., Pavuluri S.K., Cummins G., Desmulliez M.P.Y. (2020). Wireless power transfer techniques for implantable medical devices: A review. Sensors.

[6] Zhou Y., Liu C., Huang Y. (2020). Wireless Power Transfer for Implanted Medical Application: A Review. Energies.

[7] Moore J., Castellanos S., Xu S., Wood B., Ren H., Tse Z.T.H. (2019). Applications of Wireless Power Transfer in Medicine: State-of-the-Art Reviews. Ann. Biomed. Eng..

[8] Mahmood M.F., Mohammed S.L., Gharghan S.K. (2019). Ultrasound sensor-based wireless power transfer for low-power medical devices. J. Low Power Electron. Appl..

[9] Kakkar V. (2019). An Ultra Low Power System Architecture for Implantable Medical Devices. IEEE Access.

[10] Shadid R., Noghanian S. (2018). A Literature Survey on Wireless Power Transfer for Biomedical Devices. Int. J. Antennas Propag..

[11] Taalla R.V., Arefin M.S., Kaynak A., Kouzani A.Z. (2019). A review on miniaturized ultrasonic wireless power transfer to implantable medical devices. IEEE Access.

[12] Agarwal K., Jegadeesan R., Guo Y.X., Thakor N.V. (2017). Wireless Power Transfer Strategies for Implantable Bioelectronics. IEEE Rev. Biomed. Eng..

[13] Dinis H., Colmiais I., Mendes P.M. (2017). Extending the limits of wireless power transfer to miniaturized implantable electronic devices. Micromachines.

[14] Kim H., Member S., Hirayama H., Zhang R.U.I., Choi J., Member S. (2017). Review of near-field wireless power and communication for biomedical applications. IEEE Access.

[15] Lu Y., Ma D.B. (2016). Wireless power transfer system architectures for portable or implantable applications. Energies.

[16] Altawy R., Youssef A.M. (2016). Security Tradeoffs in Cyber Physical Systems: A Case Study Survey on Implantable Medical Devices. IEEE Access.

[17] Campi T., Cruciani S., De Santis V., Feliziani M. (2016). EMF Safety and Thermal Aspects in a Pacemaker Equipped with a Wireless Power Transfer System Working at Low Frequency. IEEE Trans. Microw. Theory. Tech..

[18] Liu C., Guo Y.X., Sun H., Xiao S. (2014). Design and safety considerations of an implantable rectenna for far-field wireless power transfer. IEEE Trans. Antennas Propag..

[19] Sun T., Xie X., Wang Z. (2013). Wireless Power Transfer for Medical Microsystems.

[20] Van Schuylenbergh K., Puers R. (2009). Inductive Powering: Basic Theory and Application to Biomedical Systems.

[21] Yilmaz G., Dehollain C. (2017). Wireless Power Transfer and Data Communication for Neural Implants.

[22] Zhong W., Xu D., Hui R.S.Y. (2020). Wireless Power transfer—Between Distance and Efficiency.

[23] Goldberg B.E., Everhart K., Stevens R., Babbitt N., Clemens P., Stout L. (1994). System Engineering Toolbox for Design-Oriented Engineers.

[24] Bleakley G., Lapping A., Whitfield A. Determining the right solution using SysML and model based systems engineering (MBSE) for trade studies. Proceedings of the 21st Annual International Symposium of the International Council on Systems Engineering, INCOSE 2011.

[25] Fernández J.L., Martínez Rojas J.A., Díez-Jiménez E. (2021). Modeling the Mission Dimension: The case of an intravascular medical device. Proceedings of the International Council on Systems Engineering Workshop 2021, INCOSE.

[26] Parnell G.S., Trainor T.E. Using the swing weight matrix to weight multiple objectives. Proceedings of the 19th Annual International Symposium of the International Council on Systems Engineering, INCOSE 2009.

[27] Smith E.D., Young J.S., Plattelli-Palmarinl M., Bahill A.T. (2007). Ameliorating mental mistakes in tradeoff studies. Syst. Eng..

